# Repurposing of the Tamoxifen Metabolites to Treat Methicillin-Resistant Staphylococcus epidermidis and Vancomycin-Resistant Enterococcus faecalis Infections

**DOI:** 10.1128/Spectrum.00403-21

**Published:** 2021-10-20

**Authors:** Andrea Miró-Canturri, Andrea Vila-Domínguez, Marta Caretero-Ledesma, Rafael Ayerbe-Algaba, Jerónimo Pachón, Manuel Enrique Jiménez-Mejías, Younes Smani

**Affiliations:** a Clinical Unit of Infectious Diseases, Microbiology and Preventive Medicine, Virgen del Rocío University Hospital, Seville, Spain; b Institute of Biomedicine of Seville (IBiS), Virgen del Rocío University Hospital/CSIC/University of Seville, Seville, Spain; c Department of Medicine, University of Seville, Seville, Spain; University of Calgary

**Keywords:** *Enterococcus*, Gram-positive, repurposing, *Staphylococcus*, tamoxifen, antibacterial, metabolite

## Abstract

Repurposing drugs provides a new approach to the fight against multidrug-resistant (MDR) bacteria. We have reported that three major tamoxifen metabolites, *N*-desmethyltamoxifen (DTAM), 4-hydroxytamoxifen (HTAM), and endoxifen (ENDX), presented bactericidal activity against Acinetobacter baumannii and Escherichia coli. Here, we aimed to analyze the activity of a mixture of the three tamoxifen metabolites against methicillin-resistant Staphylococcus epidermidis (MRSE) and *Enterococcus* species. MRSE (*n* = 17) and *Enterococcus* species (Enterococcus faecalis *n* = 8 and Enterococcus faecium *n* = 10) strains were used. MIC of the mixture of DTAM, HTAM, and ENDX and that of vancomycin were determined by microdilution assay. The bactericidal activity of the three metabolites together and of vancomycin against MRSE (SE385 and SE742) and vancomycin-resistant E. faecalis (EVR1 and EVR2) strains was determined by time-kill curve assays. Finally, changes in membrane permeability of SE742 and EVR1 strains were analyzed using fluorescence assays. MIC_90_ of tamoxifen metabolites was 1 mg/liter for MRSE strains and 2 mg/liter for E. faecalis and E. faecium strains. In the time-killing assays, tamoxifen metabolites mixture showed bactericidal activity at 4× MIC for MRSE (SE385 and SE742) and at 2× MIC and 4× MIC for E. faecalis (EVR1 and EVR2) strains, respectively. SE385 and EVR2 strains treated with the tamoxifen metabolites mixture presented higher membrane permeabilization. Altogether, these results showed that tamoxifen metabolites presented antibacterial activity against MRSE and vancomycin-resistant E. faecalis, suggesting that tamoxifen metabolites might increase the arsenal of drug treatments against these bacterial pathogens.

**IMPORTANCE** The development of new antimicrobial therapeutic strategies requires immediate attention to avoid the tens of millions of deaths predicted to occur by 2050 as a result of MDR bacterial infections. In this study, we assessed the antibacterial activity of three major tamoxifen metabolites, *N*-desmethyltamoxifen (DTAM), 4-hydroxytamoxifen (HTAM), and endoxifen (ENDX), against methicillin-resistant Staphylococcus epidermidis (MRSE) and *Enterococcus* spp. (E. faecalis and E. faecium). We found that the tamoxifen metabolites have antibacterial activity against MRSE, E. faecalis, and E. faecium strains by presenting MIC_90_ between 1 and 2 mg/liter and bactericidal activity over 24 h. In addition, this antibacterial activity is paralleled by an increased membrane permeability of these strains. Our results showed that tamoxifen metabolites might be potentially used as a therapeutic alternative when treating MRSE and E. faecalis strains in an animal model of infection.

## INTRODUCTION

Staphylococcus epidermidis and *Enterococcus* spp. are common health care-associated pathogens in different infections, causing significant morbidity, mortality, and/or health care costs (1–3). Glycopeptides are among the recommended treatments for the infections caused by methicillin-resistant S. epidermidis (MRSE) and ampicillin-resistant *Enterococcus* spp. (1, 4). However, the emergence of isolates with reduced susceptibility to vancomycin, teicoplanin, linezolid, and daptomycin have commonly been reported (1, 5–7). Therefore, it is important to increase the arsenal of antimicrobial agents and to find drugs active against MRSE and *Enterococcus* spp. with reduced susceptibility to glycopeptides.

Different approaches can be used to find new antibacterial agents, such as repurposing drugs. Anticancer drugs, such as tamoxifen, have demonstrated antibacterial activity against Acinetobacter baumannii, Escherichia coli, and Staphylococcus aureus (8–10). This antimicrobial activity might result from cytochrome P450-mediated tamoxifen metabolism releasing three major metabolites, *N*-desmethyltamoxifen (DTAM), 4-hydroxytamoxifen (HTAM), and endoxifen (ENDX) (11).

Few studies have investigated the activity of these metabolites against infectious agents (9, 12–15). One of them, HTAM, has been reported to act as a weak base to protect cells and mice against lethal Shiga toxin 1 (STx1) or Shiga toxin 2 (STx2) toxicosis (9) and to be active against Plasmodium falciparum and Cryptococcus neoformans var. *grubii* (13, 14). HTAM has also presented activity when used in monotherapy against Mycobacterium tuberculosis (MIC_50_ ∼2.5 to 5 mg/liter) and in combination with rifampin, isoniazid and ethambutol being the most active at 10 and 20 mg/liter of HTAM (15). Moreover, the activity of ENDX was studied against C. neoformans var. *grubii* with MIC of 4 mg/liter (14).

A previous study from our research group showed that the mixture of DTAM, HTAM, and ENDX exhibited MIC_50_ values of 8 and 16 mg/liter against clinical isolates of A. baumannii and E. coli, respectively (16), whereas their activity against Gram-positive bacteria remains unknown. The objective of this study is to investigate the activity of tamoxifen metabolites against MRSE and Enterococcus faecalis with reduced susceptibility to vancomycin.

## RESULTS

### Antimicrobial activity of tamoxifen and tamoxifen metabolites.

Tamoxifen, tamoxifen metabolites, separately and in mixture, and vancomycin were tested against clinical strains of MRSE, E. faecalis, and Enterococcus faecium. The MIC_50_ and MIC_90_ values are detailed in Table 1. The MICs of tamoxifen, tamoxifen metabolites mixture, and vancomycin for MRSE strains ranged from 2 to 4 mg/liter, 0.5 to 2 mg/liter, and 0.5 to 4 mg/liter, respectively, while those for E. faecalis strains ranged from 2 to >32 mg/liter, 1 to 2 mg/liter, and 1 to 128 mg/liter, respectively, and those for E. faecium strains ranged from 2 to 4 mg/liter, 1 to 2 mg/liter, and 0.5 to 1 mg/liter, respectively. The MIC_50_ and MIC_90_ of tamoxifen were 2 and 4 mg/liter (for MRSE strains), 8 and >32 mg/liter (for E. faecalis strains), and 4 mg/liter (for E. faecium strains). The MIC_50_ and MIC_90_ for DTAM, HTAM, and ENDX for the three pathogens ranged from 2 to 32 mg/liter. When these three metabolites were grouped together, their MIC_50_ and MIC_90_ were 1 and 2 mg/liter, respectively, for MRSE and E. faecalis and 1 and 2 mg/liter, respectively, for E. faecium. In the case of vancomycin, the MIC_50_ and MIC_90_ were 2 and 4 mg/liter (for MRSE strains), 2 and 128 mg/liter (for E. faecalis strains), and 1 mg/liter (for E. faecium strains). Of note, the checkerboard assay analysis showed that all different combinations between two tamoxifen metabolites have a slight increase in the inhibitory activity from the additive of both tamoxifen metabolites combined, with a fractional inhibitory concentration index (FICI) between 0.56 and 1 (Table 2). These results showed that the mixture of tamoxifen metabolites presented higher antibacterial activity than their prodrug tamoxifen and vancomycin against MRSE and *Enterococcus* species strains.

**TABLE 1:** MICs effective for ≥50% and ≥90% of isolates tested (MIC_50_ and MIC_90_) of tamoxifen, tamoxifen metabolites, and vancomycin for S. epidermidis and *Enterococcus* spp.^*a*^

Pathogen	*n*	TAM (mg/liter)		DTAM (mg/liter)		HTAM (mg/liter)		ENDX (mg/liter)		MET (mg/liter)		Vancomycin (mg/liter)	
		MIC_50_	MIC_90_	MIC_50_	MIC_90_	MIC_50_	MIC_90_	MIC_50_	MIC_90_	MIC_50_	MIC_90_	MIC_50_	MIC_90_
S. epidermidis	17	2	4	2	4	8	8	4	8	**1**	**1**	2	4
E. faecalis	8	8	>32	4	4	8	32	8	16	**2**	**2**	2	128
E. faecium	10	4	4	2	4	8	8	4	8	**1**	**2**	1	1

a MET: tamoxifen metabolites, 4-hydroxytamoxifen (HTAM), *N*-desmethyltamoxifen (DTAM), endoxifen (ENDX) mixture. TAM, tamoxifen; VAN, vancomycin. *Enterococcus* spp., E. faecalis and E. faecium.

**TABLE 2:** Checkerboard analysis of tamoxifen metabolites combinations against S. epidermidis SE385 and SE742 strains and E. faecalis EVR1 and EVR2 strains^*a*^

Strain	FICI (DTAM + HTAM)	FICI (DTAM + ENDX)	FICI (HTAM + ENDX)
S. epidermidis SE385	1	0.6	1
S. epidermidis SE742	0.75	0.75	1
E. faecalis EVR1	0.75	0.75	1
E. faecalis EVR2	1	0.75	0.56

a FICI, fractional inhibitory concentration index; DTAM, *N*-desmethyltamoxifen; HTAM, 4-hydroxytamoxifen; ENDX, endoxifen.

### Time-kill curves.

**FIG 1:**
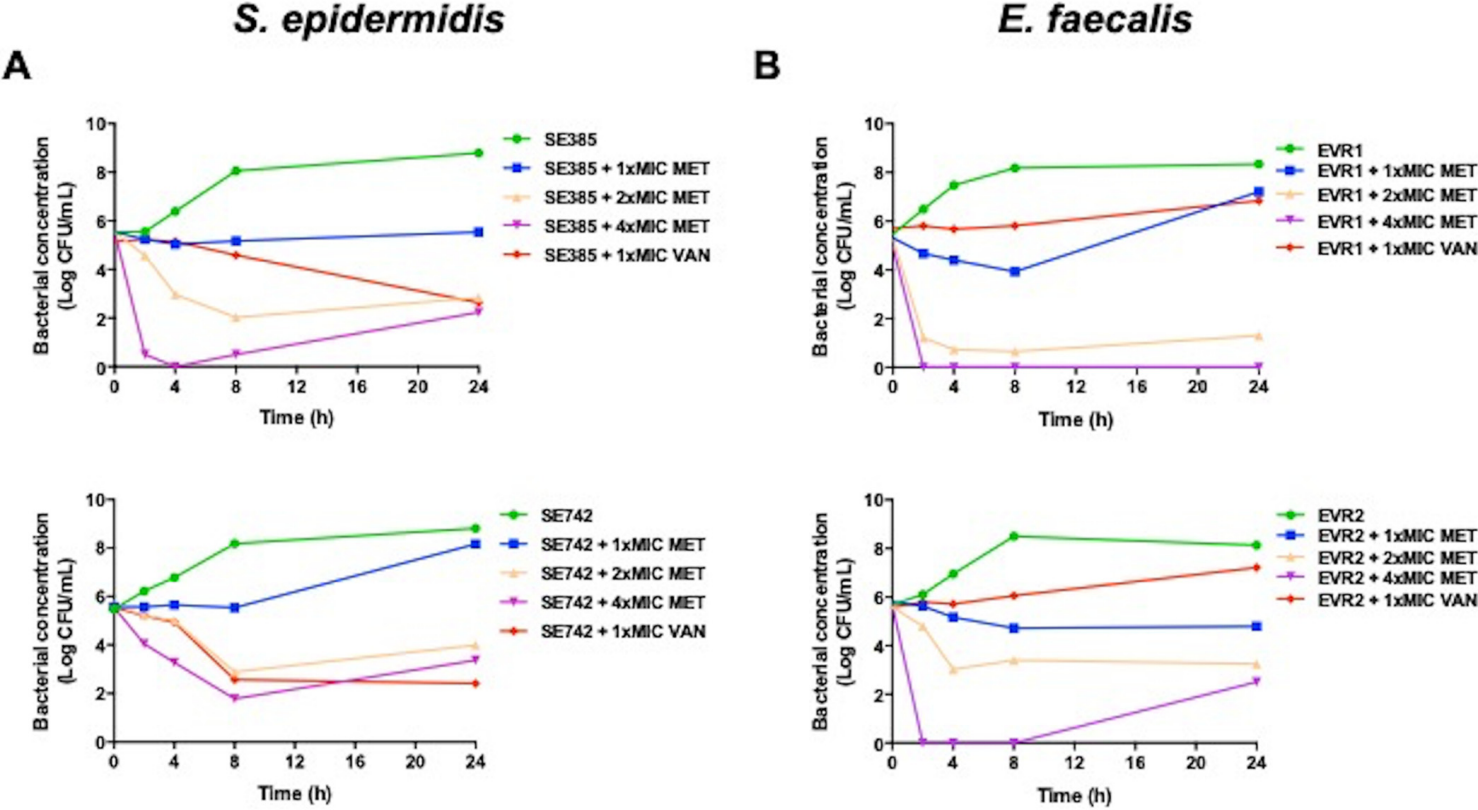
Antibacterial activity of tamoxifen metabolites at different concentrations against S. epidermidis and Enterococcus faecalis strains. Time-kill curves of S. epidermidis SE385 and SE742 strains (A) and E. faecalis EVR1 and EVR2 strains (B) in the presence of 1×, 2×, and 4× MIC tamoxifen metabolites and 1× MIC vancomycin for 24 h. MET, tamoxifen metabolites; VAN, vancomycin. Data are represented as mean from two independent experiments.

Using time-kill assays, we examined the bactericidal activity of tamoxifen metabolites and vancomycin against MRSE SE385 and SE742 strains and vancomycin-resistant E. faecalis EVR1 and EVR2 strains (Fig. 1). The MICs and MBCs of tamoxifen metabolites and vancomycin for these strains are summarized in Table 3. Tamoxifen metabolites at 4× MIC showed bactericidal activity for both MRSE strains, whereas at 1× MIC and 2× MIC they were not bactericidal (Fig. 1A). In the case of E. faecalis EVR1 and EVR2 strains, tamoxifen metabolites at 2× MIC and 4× MIC showed bactericidal activity for both strains (Fig. 1B). For SE385, SE742, and EVR2 strains, a regrowth has been particularly observed at 24 h in the presence of tamoxifen metabolites at 4× MIC. Finally, vancomycin at 1× MIC was bactericidal against SE385 and SE742 strains but not against EVR1 and EVR2 strains.

**TABLE 3:** MICs and minimal bactericidal concentrations of tamoxifen metabolites and vancomycin for S. epidermidis SE385 and SE742 and E. faecalis EVR1 and EVR2 strains^*a*^

Strain	MET (mg/liter)		VAN (mg/liter)	
	MIC	MBC	MIC	MBC
S. epidermidis SE385	1	2	4	4
S. epidermidis SE742	1	2	4	4
E. faecalis EVR1	2	4	128	>256
E. faecalis EVR2	1	2	128	>256

a MET: tamoxifen metabolites 4-hydroxytamoxifen (HTAM), *N*-desmethyltamoxifen (DTAM), endoxifen (ENDX) mixture; MBC, minimal bactericidal concentration.

### *In vitro* cytotoxicity of tamoxifen metabolites.

The study of the cytotoxicity of the tamoxifen metabolites was carried out. The percentage of cell viability on the human lung epithelial cells (A549 cells) and murine macrophages (RAW 264.7 cells) incubated for 24 h with the mixture of DTAM, HTAM, and ENDX at decreasing concentrations was determined from 400 to 0 mg/liter. Only at 400 mg/liter did this mixture show reduction in the cell viability below 50%, while the rest of the concentrations showed higher cell viability, between 83% and 100% (Table S1).

### Effect of tamoxifen metabolites on the bacterial cell membrane.

**FIG 2:**
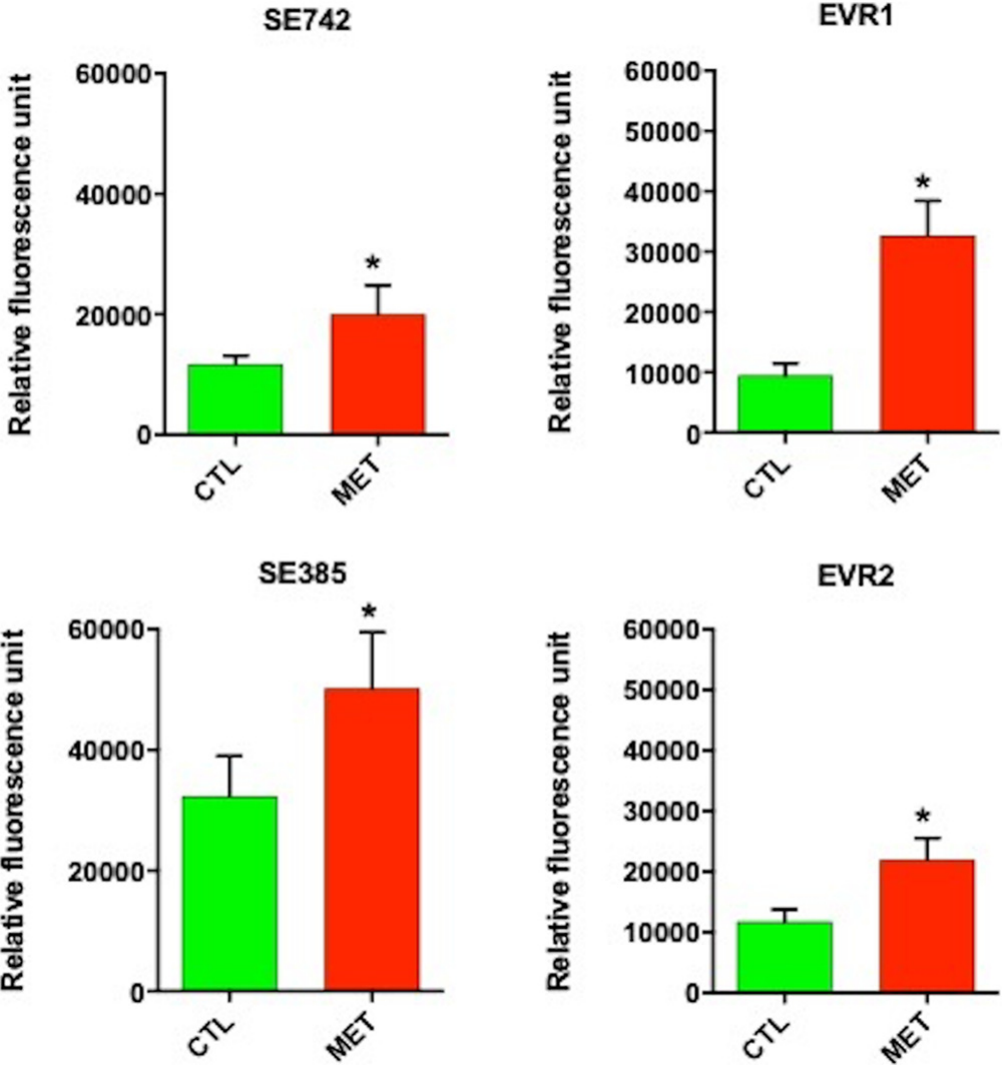
Tamoxifen metabolites effects on the bacterial permeability of S. epidermidis and E. faecalis strains. The membrane permeabilization of S. epidermidis (SE742 and SE385) and E. faecalis (EVR1 and EVR2) strains in absence and presence of tamoxifen metabolites (0.5× MIC) incubated for 10 min was quantified by Typhon Scanner. MET, the three tamoxifen metabolites together; CTL, control. *, *P* < 0.05: CTL versus MET.

In order to determine the mode of action of tamoxifen metabolites, we examined their effect on the membrane permeability of S. epidermidis (SE742 and SE385) and E. faecalis (EVR1 and EVR2) strains. The three tamoxifen metabolites mixture at 0.5× MIC significantly increased the membrane permeability of these strains, by 70.22%, 54.97%, 244.61%, and 86.6%, respectively (Fig. 2). This result suggests that tamoxifen metabolites affect the integrity of the bacterial cell wall of MRSE and vancomycin-resistant E. faecalis.

## DISCUSSION

The present study provides new data highlighting the antibacterial effect of tamoxifen metabolites against Gram-positive bacteria through the increase of bacterial membrane permeability.

The MIC_50_ of tamoxifen metabolites was 1 and 2 mg/liter against MRSE and E. faecalis, respectively. However, the MIC_50_ values obtained against Gram-negative bacteria A. baumannii and E. coli were 8 and 16 mg/liter, respectively (16). Obvious reasons for this difference could be the structural and molecular differences between the two classes of bacteria (17). Similar differences between Gram-negative and Gram-positive bacteria have been observed with other repurposed drugs, such as the statin (simvastatin), anthelmintics (niclosamide, oxycloznide, and closantel), and anti-inflammatory (celecoxib) drugs (18–20).

The antibacterial activity of tamoxifen metabolites at 4× MIC with MRSE began earlier than that of vancomycin. This result may be related to the ability of tamoxifen metabolites to eliminate staphylococcal biofilms as observed with the tamoxifen analogue, toremifene, compared with the reduced ability of vancomycin to penetrate biofilms (21, 22). In addition to the good antibacterial activity of tamoxifen metabolites at 4× MIC, a regrowth of MRSE strains and E. faecium EVR2 strain has been observed at 24 h. The MIC of tamoxifen metabolites for SE385, SE742, and EVR strains in this time-kill condition were 2, 2, and 1 mg/liter, respectively, MICs below the 4× MIC of tamoxifen metabolites concentration. Further investigations, including the determination of tamoxifen metabolites concentration during the time-kill assay, are necessary to better understand the regrowth of these strains in the presence of tamoxifen metabolites.

In this study, we showed that the three tamoxifen metabolites together produced an increase in membrane permeability of MRSE and vancomycin-resistant E. faecalis strains. It is known that the mechanism of action of tamoxifen, the prodrug of DTAM, HTAM, and ENDX in fungi, is related to the binding to calmodulin (23, 24). Additionally, Scott et al. showed that HTAM might inhibit the phospholipase D in Pseudomonas aeruginosa (25). Future studies on the mechanism of action used by tamoxifen metabolites against Gram-positive bacteria and on their therapeutic efficacy in animal experimental models of infection would be of interest. In addition to being antibacterial against Gram-positive bacteria, tamoxifen and its metabolites would have two properties that are advantageous for the treatment of bacterial infections. First, these drugs have excellent bioavailability and, therefore, can be administered orally (26), and second, they would induce the killing activity of macrophages and neutrophils similarly to that observed against Gram-negative bacteria (8).

In conclusion, these results suggest that tamoxifen metabolites are potential antimicrobial agents for use against MRSE and vancomycin-resistant E. faecalis, respectively, and they may, after further development, become a possible option for the treatment of infections by MRSE and vancomycin-resistant *Enterococcus* spp.

## MATERIAL AND METHODS

### Bacterial strains.

Seventeen MRSE and 18 *Enterococcus* species (E. faecalis *n* = 8, E. faecium *n* = 10) clinical isolates from blood cultures, characterized previously (27, 28), were used in this study. MIC susceptibility breakpoint of vancomycin for both pathogens was determined according to the standard recommendations of the Clinical and Laboratory Standards Institute (CLSI) being susceptible at ≤4 mg/liter and resistant at >4 mg/liter (29).

### Antimicrobial agents and reagents.

Standard laboratory powders of tamoxifen, HTAM, DTAM, ENDX, and vancomycin (Sigma, Spain) were used. The mixture of HTAM, DTAM, and ENDX was dissolved in dimethyl sulfoxide (DMSO) in equal concentrations.

### *In vitro* susceptibility testing.

MICs of HTAM, DTAM, and ENDX separately and in mixture, tamoxifen, and vancomycin against MRSE and *Enterococcus* species strains were determined in two independent experiments by broth microdilution assay according to CLSI guidelines (29). The initial bacterial inoculum of 5 × 10^5^ CFU/ml for each strain cultured in Mueller-Hinton Broth (MHB) (Sigma, Spain) was used in a 96-well plate (Deltlab, Spain) in the presence of HTAM, DTAM, and ENDX separately and in mixture (at same concentration), tamoxifen, and vancomycin and incubated for 24 h at 37°C. E. faecalis ATCC 29212 and S. aureus ATCC 29213 strains were used as control strains. MIC_50_ and MIC_90_, respectively, were determined.

### Checkerboard assay.

The assay was performed on a 96-well plate as described previously (30). The tamoxifen metabolite DTAM (metabolite 1), HTAM (metabolite 2), or ENDX (metabolite 3) was 2-fold serially diluted along the *x* axis, whereas the corresponding combined tamoxifen metabolite was 2-fold serially diluted along the *y* axis to create a matrix, where each well consists of a combination of both agents at different concentrations. Bacterial cultures grown overnight were then diluted in saline to 0.5 McFarland turbidity, followed by 1:50 further dilution Mueller-Hinton broth and inoculation on each well to achieve a final concentration of approximately 5.5 × 10^5^ CFU/ml. The 96-well plates were then incubated at 37°C for 24 h and examined for visible turbidity. The fractional inhibitory concentration (FIC) of metabolite 1 was calculated by dividing the MIC of metabolite 1 in the presence of metabolite 2 by the MIC of metabolite 1 alone. Similarly, the FIC of metabolite 2 was calculated by dividing the MIC of metabolite 2 in the presence of metabolite 1 by the MIC of metabolite 2 alone. The FIC index was the summation of both FIC values. FIC index values of ≤0.5, >0.5 to 1, >1 to <2, and ≥2 were interpreted as synergistic, additive, indifference, and antagonism, respectively (31). The same experiment was performed with the combination of metabolite 1 and metabolite 3 and the combination of metabolite 2 and metabolite 3.

### Time-kill kinetic assays.

Time-kill curves of MRSE SE385 and SE742 strains with a vancomycin MIC of 4 mg/liter and E. faecalis EVR1 and EVR2 strains with a vancomycin MIC of 128 mg/liter were performed in duplicate as described previously (29, 30). Initial inoculums of 5.5 × 10^5^ CFU/ml were added on 5 ml of MHB in the presence of 1×, 2×, and 4× MIC of HTAM, DTAM, and ENDX mixture and 1× MIC of vancomycin. Drug-free broth was evaluated in parallel as a control. Tubes of each condition were incubated at 37°C with shaking (180 rpm), and viable counts were determined by serial dilution at 0, 2, 4, 8, and 24 h. Viable counts were determined by plating 100 μl of control, test cultures, or the respective dilutions at the indicated times onto sheep blood agar plates (ThermoFisher, Spain). Plates were incubated for 24 h at 37°C, and after colony counts, the log_10_ of viable cells (CFU/ml) was determined. Bactericidal activity was defined as a reduction of ≥3 log_10_ CFU/ml at 24 h with respect to the initial inoculum.

### *In vitro* toxicity of the tamoxifen metabolites.

Human lung epithelial A549 cells and murine macrophages RAW 264.7 cells were incubated with the mixture of DTAM, HTAM, and ENDX (0, 50, 100, 200, and 400 mg/liter) for 24 h with 5% CO_2_ at 37°C. Prior to the evaluation of the tamoxifen metabolites cytotoxicity, A549 and RAW 264.7 cells were washed three times with prewarmed phosphate-buffered saline (PBS) 1×. Subsequently, quantitative cytotoxicity was evaluated by measuring the mitochondrial reduction activity using the 3-(4,5-dimethylthiazol-2-yl)-2,5-diphenyltetrazolium bromide (MTT) assay as described previously (32). The percentage of cytotoxicity was calculated from the absorbance at 570 nm as follows: (absorbance at 570 nm of treated cells/mean absorbance at 570 nm of untreated cells) × 100.

### Membrane permeability assays.

Bacterial suspensions (adjusted to optical density at 600 nm of 0.2) of SE742, SE385, EVR1, and EVR2 strains were placed on a 96-well plate, incubated with 0.5× MIC of tamoxifen metabolites mixture, and mixed in a solution of phosphate-buffered saline containing ethidium homodimer-1 (EthD-1; 1:500; Invitrogen, Carlsbad, CA, USA). After 10 min of incubation, fluorescence was monitored during 160 min using a Typhoon FLA 9000 laser scanner (GE Healthcare Life Sciences, Marlborough, MA, USA) and quantified with ImageQuant TL software (GE Healthcare Life Sciences, USA). Bacterial counts were obtained at the beginning and end of the experiment to ensure that the metabolite mixture did not present bactericidal activity against S. epidermidis and E. faecalis strains.

### Statistical analysis.

Group data were presented as means ± standard errors of means (SEM). Difference in membrane permeability was assessed by Student’s *t* test. The SPSS (version 23.0; SPSS Inc., Armonk, NY, USA) statistical package was used.

## References

[1] Becker K, Heilmann C, Peters G. 2014. Coagulase-negative staphylococci. Clin Microbiol Rev 27:870–926. doi:10.1128/CMR.00109-13. 25278577 PMC4187637

[2] Heilmann C, Ziebuhr W, Becker K. 2019. Are coagulase-negative staphylococci virulent? Clin Microbiol Infect 25:1071–1080. doi:10.1016/j.cmi.2018.11.012. 30502487

[3] Chiang HY, Perencevich EN, Nair R, Nelson RE, Samore M, Khader K, Chorazy ML, Herwaldt LA, Blevins A, Ward MA, Schweizer ML. 2017. Incidence and outcomes associated with infections caused by vancomycin-resistant Enterococci in the United States: systematic literature review and meta-analysis. Infect Control Hosp Epidemiol 38:203–215. doi:10.1017/ice.2016.254. 27825401

[4] Girón-González JA, Pérez-Cano R. 2003. Tratamiento de las infecciones por enterococo. Rev Clin Esp 203:482–485. doi:10.1016/S0014-2565(03)71333-2. 14563241

[5] Pfaller MA, Sader HS, Flamm RK, Castanheira M, Smart JI, Mendes RE. 2017. *In vitro* activity of telavancin against clinically important Gram-positive pathogens from 69 U.S. medical centers (2015): potency analysis by U.S. census divisions. Microb Drug Resist 23:718–726. doi:10.1089/mdr.2017.0022. 28727951 PMC5646740

[6] Santerre Henriksen A, Smart J, Hamed K. 2018. Comparative activity of ceftobiprole against coagulase-negative staphylococci from BSAC bacteraemia surveillance programme 2013-2015. Eur J Clin Microbiol Infect Dis 37:1653–1659. doi:10.1007/s10096-018-3295-6. 29876773 PMC6133033

[7] Levitus M, Rewane A, Perera TB. 2020. Vancomycin-resistant enterococci (VRE). *In* StatPearls. StatPearls Publishing, Treasure Island (FL). 30020605

[8] Miró-Canturri A, Ayerbe-Algaba R, del Toro R, Mejías ME-J, Pachón J, Smani Y. 2021. Potential tamoxifen repurposing to combat infections by multidrug-resistant Gram-negative bacilli. Pharmaceuticals 14:507. doi:10.3390/ph14060507. 34073235 PMC8230278

[9] Selyunin AS, Hutchens S, McHardy SF, Mukhopadhyay S. 2019. Tamoxifen blocks retrograde trafficking of Shiga toxin 1 and 2 and protects against lethal toxicosis. Life Sci Alliance 2:e201900439. doi:10.26508/lsa.201900439. 31243048 PMC6599968

[10] Corriden R, Hollands A, Olson J, Derieux J, Lopez J, Chang JT, Gonzalez DJ, Nizet V. 2015. Tamoxifen augments the innate immune function of neutrophils through modulation of intracellular ceramide. Nat Commun 6:8369. doi:10.1038/ncomms9369. 26458291 PMC4610010

[11] Klein DJ, Thorn CF, Desta Z, Flockhart DA, Altman RB, Klein TE. 2013. PharmGKB summary: tamoxifen pathway, pharmacokinetics. Pharmacogenet Genomics 23:643–647. doi:10.1097/FPC.0b013e3283656bc1. 23962908 PMC4084801

[12] Montoya MC, Krysan DJ. 2018. Repurposing estrogen receptor antagonists for the treatment of infectious disease. mBio 9:e02272-18. doi:10.1128/mBio.02272-18. PMC6299222 30563895

[13] Weinstock A, Gallego-Delgado J, Gomes C, Sherman J, Nikain C, Gonzalez S, Fisher E, Rodriguez A. 2019. Tamoxifen activity against Plasmodium *in vitro* and in mice. Malar J 18:378. doi:10.1186/s12936-019-3012-7. 31775753 PMC6882195

[14] Butts A, Koselny K, Chabrier-Rosello Y, Semighini CP, Brown JC, Wang X, Annadurai S, DiDone L, Tabroff J, Childers WE, Jr, Abou-Gharbia M, Wellington M, Cardenas ME, Madhani HD, Heitman J, Krysan DJ. 2014. Estrogen receptor antagonists are anti-cryptococcal agents that directly bind EF hand proteins and synergize with fluconazole in vivo. mBio 5:e00765-13. doi:10.1128/mBio.00765-13. 24520056 PMC3950514

[15] Chen FC, Liao YC, Huang JM, Lin CH, Chen YY, Dou HY, Hsiung CA. 2014. Pros and cons of the tuberculosis drugome approach-an empirical analysis. PLoS One 9:e100829. doi:10.1371/journal.pone.0100829. 24971632 PMC4074101

[16] Miró-Canturri A, Ayerbe-Algaba R, Vila-Domínguez A, Jiménez-Mejías ME, Pachón J, Smani Y. 2021. Repurposing of the tamoxifen metabolites to combat infections by multidrug-resistant Gram-negative bacilli. Antibiotics 10:336. doi:10.3390/antibiotics10030336. 33810067 PMC8004611

[17] Brown L, Wolf JM, Prados-Rosales R, Casadevall A. 2015. Through the wall: extracellular vesicles in Gram-positive bacteria, mycobacteria and fungi. Nat Rev Microbiol 13:620–630. doi:10.1038/nrmicro3480. 26324094 PMC4860279

[18] Thangamani S, Mohammad H, Abushahba MF, Hamed MI, Sobreira TJ, Hedrick VE, Paul LN, Seleem MN. 2015. Exploring simvastatin, an antihyperlipidemic drug, as a potential topical antibacterial agent. Sci Rep 5:16407. doi:10.1038/srep16407. 26553420 PMC4639749

[19] Rajamuthiah R, Fuchs BB, Conery AL, Kim W, Jayamani E, Kwon B, Ausubel FM, Mylonakis E. 2015. Repurposing salicylanilide anthelmintic drugs to combat drug resistant *Staphylococcus aureus*. PLoS One 10:e0124595. doi:10.1371/journal.pone.0124595. 25897961 PMC4405337

[20] Thangamani S, Younis W, Seleem MN. 2015. Repurposing celecoxib as a topical antimicrobial agent. Front Microbiol 6:750. doi:10.3389/fmicb.2015.00750. 26284040 PMC4517059

[21] De Cremer K, Delattin N, De Brucker K, Peeters A, Kucharíková S, Gerits E, Verstraeten N, Michiels J, Van Dijck P, Cammue BPA, Thevissen K. 2014. Oral administration of the broad-spectrum antibiofilm compound toremifene inhibits *Candida albicans* and *Staphylococcus aureus* biofilm formation *in vivo*. Antimicrob Agents Chemother 58:7606–7610. doi:10.1128/AAC.03869-14. 25288093 PMC4249581

[22] Sing R, Ray P, Das A, Sharma M. 2010. Penetration of antibiotics through *Staphylococcus aureus* and *Staphylococcus epidermidis* biofilms. J Antimicrob Chemother 65:1955–1958. doi:10.1093/jac/dkq257. 20615927

[23] Dolan K, Montgomery S, Buchheit B, DiDone L, Wellington M, Krysan DJ. 2009. Antifungal activity of tamoxifen: *in vitro* and *in vivo* activities and mechanistic characterization. Antimicrob Agents Chemother 53:3337–3346. doi:10.1128/AAC.01564-08. 19487443 PMC2715577

[24] Butts A, Martin JA, DiDone L, Bradley EK, Mutz M, Krysan J. 2015. Structure-activity relationships for the antifungal activity of selective estrogen receptor antagonists related to tamoxifen. PLoS One 10:e0125927. doi:10.1371/journal.pone.0125927. 26016941 PMC4446328

[25] Scott SA, Spencer CT, O’Reilly MC, Brown KA, Lavieri RR, Cho C-H, Jung D-I, Larock RC, Brown HA, Lindsley CW. 2015. Discovery of desketoraloxifene analogues as inhibitors of mammalian, *Pseudomonas aeruginosa*, and nape phospholipase d enzymes. ACS Chem Biol 10:421–432. doi:10.1021/cb500828m. 25384256 PMC4336625

[26] Morello KC, Wurz GT, DeGregorio MW. 2003. Pharmacokinetics of selective estrogen receptor modulators. Clin Pharmacokinet 42:361–372. doi:10.2165/00003088-200342040-00004. 12648026

[27] Domínguez-Herrera J, Docobo-Pérez F, López-Rojas R, Pichardo C, Ruiz-Valderas R, Lepe JA, Pachón J. 2012. Efficacy of daptomycin versus vancomycin in an experimental model of foreign-body and systemic infection caused by biofilm producers and methicillin-resistant *Staphylococcus epidermidis*. Antimicrob Agents Chemother 56:613–617. doi:10.1128/AAC.05606-11. 22123684 PMC3264234

[28] Vila Domínguez A, Ayerbe-Algaba R, Miró Canturri A, Rodríguez-Villodres A, Smani Y. 2020. Antibacterial activity of colloidal silver against Gram-negative and Gram-positive bacteria. Antibiotics 9:36. doi:10.3390/antibiotics9010036. PMC7167925 31963769

[29] Clinical and Laboratory Standards Institute. 2019. Performance standards for antimicrobial susceptibility testing, 29th ed. CLSI, Wayne, PA, USA.

[30] Miró-Canturri A, Ayerbe-Algaba R, Villodres ÁR, Pachón J, Smani Y. 2020. Repositioning rafoxanide to treat Gram-negative bacilli infections. J Antimicrob Chemother 75:1895–1905. doi:10.1093/jac/dkaa103. 32240294

[31] EUCAST. 1998. Methods for the determination of susceptibility of bacteria to antimicrobial agents. Terminology Clin Microbiol Infect 4:291–296. doi:10.1111/j.1469-0691.1998.tb00061.x. 11864348

[32] Ayerbe-Algaba R, Bayó N, Verdú E, Parra-Millán R, Seco J, Teixidó M, Pachón J, Giralt E, Smani Y. 2021. AOA-2 derivatives as outer membrane protein a inhibitors for treatment of Gram-negative bacilli infections. Front Microbiol 12:634323. doi:10.3389/fmicb.2021.634323. 33643267 PMC7907166

