# Supplementary material for: Repurposing of the Tamoxifen Metabolites to Treat Methicillin-Resistant Staphylococcus epidermidis and Vancomycin-Resistant Enterococcus faecalis Infections
Source: Microbiol Spectr. 2021 Oct 20;9(2):e00403-21. doi: 10.1128/Spectrum.00403-21 (PMC8528103; doi:10.1128/Spectrum.00403-21)
Supplement: SUPPLEMENTAL FILE 1 — Supplemental material. Download SPECTRUM00403-21_Supp_1_seq2.pdf, PDF file, 0.3 MB [file spectrum00403-21_supp_1_seq2.pdf]

Supplementary data

Table S1. Effect of the mixture of DTAM, HTAM and ENDX on the cell viability.

Mixture concentrations	A549 cells	RAW 264.7
0 mg/L	100.00% ± 3.05%	100.00% ± 4.82%
50 mg/L	100.56% ± 9.18%	99.91% ± 1.68%
100 mg/L	98.53% ± 5.29%	92.03% ± 0.35%
200 mg/L	87.93% ± 3.26%	82.64% ± 0.20%
400 mg/L	38.26% ± 13.64%	47.62% ± 1.27%
